# Subjective Well-Being in Organizations: Effects of Internal Ethical Context and Ethical Leadership

**DOI:** 10.3390/ijerph20054451

**Published:** 2023-03-02

**Authors:** Rita Paralta, Eduardo Simões, Ana Patrícia Duarte

**Affiliations:** 1 Instituto Universitário de Lisboa (ISCTE-IUL), 1649-026 Lisbon, Portugal; 2 Centro de Estudos sobre a Mudança Socioeconómica e o Território (DINÂMIA’CET), ISCTE-IUL, 1649-026 Lisbon, Portugal; 3 Business Research Unit (BRU-IUL), ISCTE-IUL, 1649-026 Lisbon, Portugal

**Keywords:** internal ethical context, ethical infrastructure, corporate social responsibility, subjective well-being, ethical leadership, ethics codes, ethics programs

## Abstract

The literature rarely addresses the possible effects of organizations’ internal ethical context on their employees’ subjective well-being, that is, people’s evaluation of their lives based on positive and negative emotional experiences and perceived life satisfaction. This study explored how internal ethical context’s components—specifically ethics codes, ethics programs’ scope and perceived relevance, and perceived corporate social responsibility practices—are related to workers’ subjective well-being. Ethical leadership’s possible leveraging of ethical context variables’ effect on subjective well-being was also examined. The data were collected from 222 employees from various organizations in Portugal using an electronic survey. The results from multiple regression analyses indicate that organizations’ internal ethical context positively affects employees’ subjective well-being. This impact is mediated by ethical leadership, suggesting that leaders play a crucial role in highlighting and embodying their organization’s ethical norms and orientation, thereby directly influencing their staff members’ subjective well-being.

## 1. Introduction

When various ethical scandals became public in recent years, most organizations reviewed their standards to reduce unethical behavior [1]. These efforts indicate that managers acknowledge that wrongdoing contributes to high financial costs, damages reputation, undermines employees’ morale, and even threatens organizations’ survival [2]. The process of the institutionalization of ethics [3] translates into programs that include explicit elements such as codes or regulations and implicit elements, for example, organizational culture or incentive systems [4]. Ethics programs are thus a fundamental part of internal ethical contexts, that is, “organizational factors that stimulate managers and employees to behave ethically” [5] (p. 2).

As predicted by ethical impact theory [6], at an individual level, unethical behavior in the workplace, such as bullying and discrimination, can harm worker experience and result in decreasing levels of physical and psychological well-being. Conversely, ethical contexts can positively affect organizational outcomes and processes. For instance, a highly ethical climate is an implicit element of internal ethical contexts associated with reduced turnover and stronger work involvement [7,8]. However, the specific effects of organizations’ internal ethical context on their staff members’ psychological well-being in all social settings (i.e., in and outside the workplace) have been overlooked.

Subjective well-being (SWB) refers to how individuals evaluate their lives. As people spend a large part of their lives at work, workers’ experience and personal outcomes in organizations, such as job satisfaction [9] or affective well-being at work [10], can have a strong impact on individuals’ assessment of their happiness and well-being in life [11]. Some of the few prior studies of internal ethical contexts’ effect on SWB have found proxy negative associations between these two sets of variables. For example, recurrent unethical behavior in organizations is negatively associated with job satisfaction and commitment [12], and this behavior’s prevalence can contribute to depression, anxiety, and other health problems [13]. However, with a few notable exceptions [14,15], researchers have infrequently examined internal ethical context factors’ possible impacts on SWB.

The present study sought to expand the existing knowledge on this topic by investigating the association between relevant elements of organizations’ internal ethical context and SWB. More specifically, the purpose of this study was (i) to assess how internal ethical context’s components, namely ethics codes, ethics programs’ scope, and its perceived relevance, and corporate social responsibility practices are related to workers’ SWB and (ii) to examine ethical leadership intervention as a mediator in the relationship between internal ethical context’s components and SWB. Given this focus, the remainder of this paper is structured as follows. The next section presents a review of the pertinent literature and theoretical framework underlying the research model as well as the hypotheses’ development. In the third and fourth sections, the methodology is described, including sample, procedures, and measures, after which the statistical analyses and results are presented. The final section discusses the findings’ theoretical and practical implications.

## 2. Literature Review and Hypothesis Development

### 2.1. Subjective Well-Being

SWB refers to the extent to which a person is satisfied with his or her life. The concept encompasses both an affective dimension related to positive and negative emotional experiences’ frequency and a cognitive dimension concerning assessments of overall life satisfaction [16], often referred to as “happiness” or “satisfaction with life”. Although these two dimensions are strongly correlated, they differ at a theoretical level, so they can be measured and studied independently [17].

Negative feelings refer to the subjective experience of negative emotional states such as shame, sadness, anger, guilt, envy, and anxiety, which lead to negative effects on people’s daily lives, such as stress and increased propensity for health problems [18].

SWB’s cognitive dimension is also related to satisfaction with life so that, in an ideal world, each person can evaluate his or her life as “good” [19]. This dimension encompasses an appreciation of what is expected versus accomplished in past and current moments in different areas of life such as work, family, leisure, health, and finance.

Individuals with a high level of SWB often feel positive emotions and rarely have negative emotional experiences and frequently express satisfaction with specific and general aspects of life [20]. People with a high level of SWB tend to have a positive influence on their social environment, as they are more likely to exhibit socially desirable behavior and perform altruistic or social activities (e.g., volunteering and charity work) than individuals with lower levels [16]. The existing evidence suggests that SWB is also an advantageous factor in the workplace since happy workers are more likely to be successful and actively involved in various aspects of organizational outcomes and processes. These employees tend to help their work colleagues and more often exhibit desirable social behavior [21]. In short, a high level of SWB not only has social benefits but also favors success in the workplace, including a lasting positive influence on job performance [22].

### 2.2. Organizations’ Internal Ethical Context and SWB

Issues such as ethics and quality of life in the workplace have gained greater prominence in recent years as ways to contribute positively to organizational sustainability and overall performance [23,24,25]. In general, people who work in organizations that value ethical approaches to challenges (e.g., codes of ethics and ethics training programs) tend to be more satisfied [7,26,27]. Organizations that are concerned about their operations’ ethical quality seek to create environments conducive to incorporating ethical guidelines into decision-making processes and influencing their employees’ behavioral and moral development [28,29].

Principle statements and standards are key elements of an organization’s ethical infrastructure, which can be defined as a set of procedures and mechanisms focused on stimulating attitudes that support ethical values [30]. Ethical infrastructure is a more inclusive construct than the notion of an ethical program. The former comprises formal and informal components such as communication systems to transmit ethical principles, surveillance systems to monitor adherence to those principles, and mechanisms that reward ethical behavior and punish unethical conduct [31].

Codes of ethics are at the core of the ethical infrastructure, providing formal rules for employees’ ethical conduct and including injunctions to prevent unethical behavior. This tends to occur more frequently in organizations without an ethics program compared to those that have one [32], although codes of ethics alone are not enough to guarantee organizational ethics [33], and the codes’ impact is difficult to assess empirically. Early research on ethical codes of conduct’s effects on behavior [34] found that no significant impact existed, but later studies considered variables that can influence compliance with codes [35]. The most recent results suggest that codes have an overall positive effect on employees’ ethical behavior and, more recently, commitment and organizational performance [36].

To be effective, codes of ethics rely on ethics programs’ design and dynamics, which are important determinants of how strongly ethical infrastructure can influence individual and group behavior within organizations. Ethics programs’ scopes vary depending on organizations’ socioeconomic and cultural backgrounds [37]. That is, programs can encompass diverse components such as a code of ethics, training, communication plans, monitoring and audits, accountability policies, a hotline for reporting unethical behavior, and incentive policies. Some components (e.g., monitoring) can be more essential [38], but the available evidence points to ethics programs’ overall scope as one of the most important variables in terms of explaining programs’ influence on employees. The greater the number of components included, the less frequent unethical behavior becomes [32].

The importance that employees give to ethics programs depends on each individual’s perception of the surrounding ethical infrastructure, so these programs must be visible and prominent. A multifaceted ethical infrastructure also significantly increases moral awareness and has a positive impact on intentions to engage in ethical behavior [39], but each program component needs to be fully understood by organizations’ staff members. For instance, to reduce unethical behavior, codes of ethics must influence employees’ coping appraisal process, thereby ensuring that they are clearly aware of the relevant code’s contents and ways to deal with its rules [38,40], namely how to translate norms into personal actions. Staff members’ ethical behavior and perceptions are influenced by the way in which organizations use their ethical infrastructure and raise its profile as well as by managers’ behavioral consistency [41,42].

In short, strong ethical infrastructure tends to influence employees’ beliefs regarding ethics’ importance. These, in turn, are positively associated with some indicators of SWB: Promislo and colleagues [6], based on ethical impact theory, found that ethical organizational context is negatively related to work-related stress and positively related to psychological well-being. Two recent studies have made this association even clearer, showing that organizations’ ethical context can favor workers’ well-being via positive workplace experiences [14] and reduced work-related stress [15]. Based on the literature on bullying, discrimination, and injustice, Promislo and colleagues’ ethical impact theory [6] proposes that all types of unethical behavior, from minor deviance to large and impactful decisions, can impact workers’ SWB. This is so because people react emotionally to unethical situations with three primary mechanisms explaining decreased well-being: stress, trauma, and poor health behaviors. Given that a strong internal ethical context is associated with decreased occurrence of unethical behaviors in the organization [28], it should increase workers’ perception of positive organizational ethics. Therefore, a positive relationship between the internal ethical context’s components and workers’ SWB is expected. Drawing on these previous results and inferences, the present study’s first three hypotheses posited the following:

**H1a.**  *Codes of ethics or similar guidelines have a positive relationship with SWB*.

**H1b.**  *In organizations with formal codes of ethics or similar guidelines, ethics programs’ scope—as defined by their number of elements—is positively associated with SWB*.

**H1c.**  *Ethical programs’ perceived relevance has a positive relationship with SWB*.

In this paper, it is proposed that corporate social responsibility (CSR) can be an ally of the organization’s internal ethical context. CSR refers to the degree to which organizations maximize the creation of shared value for stakeholders (e.g., investors, employees, suppliers, customers, and society at large). CSR has several dimensions including economic, social, and community and/or environmental [43,44]. Although CSR practices are not necessarily a component of ethics programs, it can be understood as an implicit component that favors ethical behavior and as taking advantage of ethical infrastructure systems.

Organizations’ CSR performance influences their employees’ attitudes and behavior [45,46]. People usually prefer to work in organizations that encourage employee initiatives and reward social responsibility activities because these practices improve organizations’ image [47]. In this way, CSR increases organizational commitment and trust [48,49], job satisfaction and work engagement [50,51], and overall quality of life [52] and lowers work-related stress [15].

In addition, socially responsible human resources management practices predict workers’ generic well-being [53]. Organizational benevolence (i.e., employers or their managers’ perceived good and honest intentions) is also positively linked to employees’ well-being [54]. The above findings were translated into the current research’s next hypothesis:

**H1d.**  *Perceived CSR practices are positively associated with SWB*.

### 2.3. Ethical Leadership as a Mediator of the Relationship berween Organizations’ Internal Ethical Context and SWB

Leaders’ support is an essential condition for ethical organizational conduct and social performance [7]. As “actions speak louder than words”, specific features of leadership practices, such as leaders’ public behavior, strongly determine employees’ ethical behavior. In this context, leadership can be defined as a process of influencing a group to achieve the desired common goals [55]. Ethical leadership, in turn, constitutes on-going practices that affect followers’ ethical conduct in ways that help them attain organizational ethics goals [56,57].

Some of the leaders’ personal characteristics (e.g., high conscientiousness and low neuroticism) are positively related to ethical leadership, but just being a morally upright person is not enough to be perceived as an ethical leader [58]. Ethical leadership entails an ability to define organizations’ ethical tone, that is, to find ways to direct staff members’ attention to ethics and support explicitly and publicly the ethical principles that can guide employees’ behavior and decisions [59]. Leaders must thus share power, give a voice to followers, ensure fair treatment, and promote discussions of workplace ethics [60,61]. However, the very essence of ethical leadership is demonstrating appropriate conduct through personal actions and interpersonal relationships, which must be transmitted to employees through bidirectional communication, reinforcement, and decision making [56,62].

Some contextual characteristics have also been identified as relevant for the development and maintenance of ethical leadership [31,63], including the organizational ethical infrastructure. This is so because their components set the rules and regulations as well as expectations regarding adequate ethical conduct and signal for the leader and remaining organizational members how to behave regarding ethical issues. Moreover, they may create an atmosphere of increased awareness of ethical issues and a working context in which ethical orientations are particularly salient and the focus of leaders’ and employees’ attention [63]. These formal guidelines and informal elements will thus encourage the adoption of an ethical leadership style.

Leaders who act as role models and are guided by ethical values, altruism, honesty, and fairness are more interested in supporting followers, which generates improved worker experience translated into greater satisfaction, commitment, and involvement in the workplace [58,64,65,66]. Conversely, ethical leadership is negatively related to cynicism [61], turnover intentions [67], and counterproductive behavior [57]. In general, a positive association has been found between ethical leadership and attitudes related to employees’ well-being, such as job satisfaction, satisfaction with leaders, optimism, commitment, and decreased emotional exhaustion [60,65,68,69,70].

Other ethical leadership outcomes are associated with SWB in diverse ways. For example, this leadership style promotes knowledge sharing among employees through the mediating effect of SWB [71]. Ethical leadership also has a positive influence on SWB that is, in turn, mediated by perceived justice [72] and workers’ voice behavior [73]. Additionally, SWB is a mediator in the relationship between ethical leadership and helping behavior, with a compensation effect. That is, SWB has a stronger impact when employees perceive human resource management practices as poor [74].

Prior studies’ results provide strong support for the conclusion that ethical leadership can play a mediating role in the relationship between internal ethical context and SWB. Being guided and stimulated by their organization’s internal ethical context, leaders might increasingly develop a stronger ethical stance that includes not only transmitting their organization’s ethics orientation to employees and thus diminishing ethical ambiguity [75] but also treating them fairly and providing support [26,27] and other resources to deal with diverse job situations, thus diminishing job-related stress [6]. This will translate into enhanced SWB. The above findings were incorporated into the present research’s last hypotheses:

**H2a.**  *Ethical leadership mediates the relationship between the existing code of ethics or similar guidelines and SWB*.

**H2b.**  *Ethical leadership mediates the relationship between the ethics program’s scope and SWB*.

**H2c.**  *Ethical leadership mediates the relationship between ethical programs’ perceived relevance and SWB*.

**H2d.**  *Ethical leadership mediates the relationship between perceived CSR practices and SWB*.

**Figure 1:**
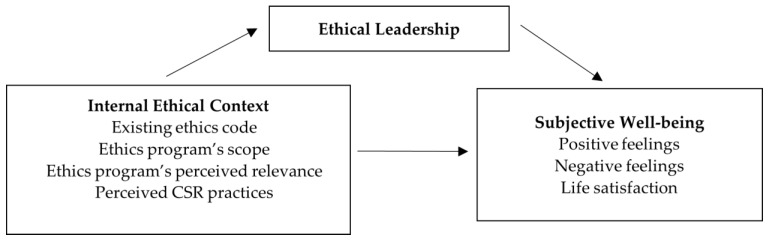
Conceptual model.

Figure 1 shows the resulting conceptual model, including the variables under analysis and the possible relationships among them.

## 3. Materials and Methods

### 3.1. Procedures and Sample

To enable an empirical examination of the research model, a quantitative, cross-sectional study was developed, following both the ethical guidelines of the Declaration of Helsinki and the research team’s university. Data were collected from a sampling frame of employees from different organizations in Portugal using an electronic survey. To participate in the survey, respondents had to meet the inclusion criteria of an age of eighteen years old or above and being employed at the time of the survey. Participation in the study was voluntary, and respondents’ anonymity and data confidentiality were guaranteed in the informed consent form. The latter also provided information about the research’s general aims and instructions on how to fill out the questionnaire. Besides collecting sociodemographic data, the items included measures for all the selected variables.

The obtained non-probabilistic convenience sample of 222 participants included 143 females (64.4%). The respondents’ ages ranged between 18 and 63 years old (mean = 37.0; standard deviation (SD) = 12.8 years). Most participants had a higher education degree (67.0%). Concerning job tenure, the respondents had a mean tenure of 10.6 years in their current organization (SD = 10.6 years; minimum = 0.1; maximum = 42 years). Most respondents worked for a privately held organization (84.7%). Regarding the organizations’ size, 51.3% were small- and medium-sized organizations (i.e., up to 250 workers), 26.6% were large (i.e., 250–500 employees), and 22.1% were very large (i.e., more than 500 employees).

### 3.2. Variables and Measures

Internal ethical context was operationalized using four variables. The existing formal code of ethics (predictor variable 1) was assessed with a dichotomous question: “Does the organization where you work have a code of ethics, that is, a formal document that articulates the organization’s values and norms of conduct or something similar?” The respondents answered either “no” (0) or “yes” (1) [32,41,42].

The ethics program’s scope (predictor variable 2) was measured by five additional items focused on different program components in organizations with a code of ethics [30,40] (i.e., ethics code training, clear rules for sanctions for misconduct, an anonymous and confidential “hotline” on ethical issues, monitoring of compliance with the ethics code, and a manager responsible for the code). These items included the following examples: “Training is provided to employees on the code of ethics or similar guidelines” and “Clear rules are provided for sanctions in case of alleged misconduct”. Responses were given as “no” (0) or “yes” (1). Following standard procedures [32,42], a composite variable was created by adding together the number of ethics program elements reported after a code of ethics’ existence was confirmed. The values could vary between 1 for only the code of ethics’ presence and 6 for an existing ethics code plus the full five elements.

Ethics programs’ perceived relevance (predictor variable 3) was measured using three items (Cronbach’s alpha (α) = 0.72) developed by Simões and colleagues [42], namely “Employees are aware of the existing code of ethics or similar guidelines in their organization”, “Workers who violate the standards established by the code are investigated and punished”, and “The different department heads of the organization play an active role in monitoring employees’ compliance with the code of ethics”. Responses were provided on a 5-point Likert scale (1 = “totally disagree”; 5 = “totally agree”).

Perceived CSR practices (predictor variable 4) was assessed with seven items (α = 0.86) previously validated [42,76], focusing on socially responsible practices involving workers (i.e., “This organization promotes equality between men and women”; “…stimulates employees’ occupational training”; “…fulfills labor laws”; “…guarantees job security”; “…Promotes work-family balance”; “…supports professional integration of the disabled”; and “…develops internal rules that guide employees’ professional behavior”). Responses were provided on a 5-point Likert scale (1 = “totally disagree”; 5 = “totally agree”).

Ethical leadership (mediating variable) was measured with the well-known Ethical Leadership Scale [56], which consists of 10 items (α = 0.95). This scale assesses employees’ opinions of their direct manager’s behavior (e.g., “My direct manager disciplines employees who violate ethical principles”). Responses were provided on a 5-point Likert scale (1 = “totally disagree”; 5 = “totally agree”).

SWB (criterion variable) was operationalized using three well-established indicators: positive feelings, negative feelings, and life satisfaction. Two different measurement scales were used: the Satisfaction with Life Scale (SWLS) and the Scale of Positive and Negative Experience (SPANE). The SWLS [77] was developed to quantify SWB’s cognitive dimension. This scale comprises five items (α = 0.84) (e.g., “In many ways, my life is close to my ideals”). Answers were provided on a 7-point Likert scale (1 = “totally disagree”; 7 = “totally agree”). The SPANE [78] is a tool to assess SWB’s affective dimension, consisting of 12 items, of which 6 items (α = 0.90) measure the frequency of positive feelings and experiences (e.g., “well”). The remaining items (α = 0.82) evaluate the frequency of negative feelings and experiences (e.g., “sad”). Responses were given on a 5-point Likert scale (1 = “never”; 5 = “always”).

### 3.3. Discriminant and Convergent Validity

The current research had a cross-sectional design, collecting data from a single source for all constructs at a single moment in time, so common method variance (CMV) could weaken the results’ validity [79,80]. Three techniques were used to check whether the questionnaire’s items captured distinct constructs as opposed to creating common source bias. Harman’s single-factor test was run first [80]. Exploratory factor analysis without rotation was conducted, revealing that the first factor measured accounts for only 11.94% of the total variance (64.94%).

Next, confirmatory factor analyses were performed. The six-factor model has fit indices within the recommended values (chi-square (χ^2^) = 1036.533; degrees of freedom (df) = 614; *p* < 0.001; χ^2^/df = 1.688; root mean square error of approximation (RMSEA) = 0.06; Tucker–Lewis index (TLI) = 0.91; comparative fit index (CFI) = 0.92). In contrast, the single-factor model presents a poor fit to the data (i.e., χ^2^ = 2972.561; df = 629; *p* < 0.001; χ^2^/df = 4.726; RMSEA = 0.13; TLI = 0.50; CFI = 0.53) [81,82].

Finally, average variance extracted (AVE) values were estimated and compared to the squared correlations between all pairs of variables, as suggested by Fornell and Larcker [83] (Table 1). The present results reveal that the AVE values are greater than the shared variance between variables. Overall, these statistics suggest that the constructs show discriminant validity and that no serious CMV is present in the results.

Regarding convergent validity, the composite reliability (CR) estimates (i.e., from 0.76 to 0.95) fall above the recommended cut-off point of 0.70 [84]. The AVE values estimated for perceived CSR (0.48) and negative feelings (0.49) are slightly below the threshold of 0.50 proposed by Fornell and Larcker [83]. As noted by the cited authors [83], AVE is a more conservative estimate of convergent validity than CR, and based on CR alone, researchers can “conclude that the convergent validity of the construct is adequate, even though more than 50% of the variance is due to error” (p. 46).

Because the present model’s constructs have CR values well above the recommended level, their convergent validity was considered acceptable, and the data analysis was continued. Given that the different measures revealed good levels of reliability, discriminant, and convergent validity (see Table 1), a composite score for each variable was calculated for each respondent by averaging the pertinent set of items, which was then used in subsequent analyses.

## 4. Results

Table 1 presents the means, SDs, CRs, AVEs, correlations, and squared correlations between the variables, showing that most but not all the variables significantly correlate with each other. The main exception is an existing ethics code, which is not significantly related to any SWB indicator. In addition, the correlation coefficients of the affective SWB variables (i.e., positive feelings, negative feelings, and life satisfaction) are statistically nonsignificant with regard to the respondents’ sociodemographic characteristics, namely gender, age, education, and job tenure. Employer type and size are also not significantly correlated with SWB indicators.

PROCESS macro for IBM SPSS version 26 software [85] was used to test the hypotheses. The first hypothesis proposed that organizations’ internal ethical context positively affects their employees’ SWB. Hypothesis H1 was divided into four sub-hypotheses referring to each of the four predictor variables examined. These were an existing ethics code, the ethics program’s scope, the ethics program’s perceived relevance, and perceived CSR practices.

The total effects reported in the next tables reveal that the internal ethical context factors produce dissimilar results for the three SWB indicators. An existing ethics code has no significant effect on the frequency of positive feelings (non-standardized coefficient (B) = 0.18; not significant (n.s.)), negative feelings (B = −0.81; n.s.), or life satisfaction (B = −0.13; n.s.) (Table 2). Therefore, hypothesis H1a was not supported.

The ethics program’s scope significantly reduces the frequency of negative feelings (B = −0.38; *p* < 0.05) but has no impact on the frequency of positive feelings (B = 0.32; *p* < 0.06; marginally significant) or satisfaction with life (B = 0.04; n.s.) (Table 3). These results provide partial support for hypothesis H1b.

The ethics program’s perceived relevance is significantly related to more frequent positive feelings (B = 1.03; *p* < 0.001) and less frequent negative feelings (B = −1.17; *p* < 0.001). However, this variable does not have a significant effect on life satisfaction (B = 0.11; n.s.) (Table 4). Thus, hypothesis H1c received partial support.

Perceived CSR has a significant impact on all SWB indicators, as this factor is associated with more frequent positive feelings (B = 2.08; *p* < 0.001) and higher life satisfaction (B = 0.27; *p* < 0.01) as well as less frequent negative feelings (B = −1.17; *p* < 0.001) (Table 5). Hypothesis H1d was, therefore, supported. Overall, the findings provide partial support for hypothesis H1 regarding the effect of organizations’ internal ethical context on their employees’ SWB. Some contextual factors have a significant effect on workers’ reported frequency of positive and negative feelings in the workplace and these individuals’ satisfaction with life.

Hypothesis H2 proposed that ethical leadership mediates the relationship between internal ethical contexts’ components and employees’ SWB. That is, their perception of internal ethical context elements should have a positive association with higher levels of perceived ethical leadership, which then should be related to higher SWB. This second hypothesis was again divided in sub-hypotheses to test for each context factor’s specific effect.

Regarding an existing ethics code, Table 2 shows that ethical leadership completely mediates the relationship between this contextual factor and the three well-being indicators, namely positive feelings (B = 0.71; 95% confidence interval (CI) = 0.22, 1.30), negative feelings (B = −0.48; 95% CI = −0.98, −0.12), and life satisfaction (B = 0.12; 95% CI = 0.02, 0.26). While an existing ethics code has no significant total effect on SWB indicators, the indirect effects are statistically significant. This result supports the assumption that an existing ethics code contributes to increased well-being only through strong ethical leadership, which then has a positive effect on employees’ SWB, indicating that only a completely indirect association exists.

The results for the ethics program’s scope indicate two significant mediation effects (Table 3): one for positive feelings (B = 0.17; 95% CI = 0.05, 0.34) and another for life satisfaction (B = 0.03; 95% CI = 0.00, 0.07). The ethics program’s scope thus contributes to more frequent positive feelings and greater life satisfaction among workers via ethical leadership. The effect on negative feelings is in the expected negative direction, but the impact is statistically nonsignificant (B = 0.08; 95% CI = −0.20, 0.01).

The findings for the ethics program’s relevance reveal that ethical leadership mediates the relationship between this variable and the three SWB indicators, namely positive feelings (B = 0.52; 95% CI = 0.26, 0.86), negative feelings (B = −0.31; 95% CI = −0.62, −0.08), and life satisfaction (B = 0.09; 95% CI = 0.01, 0.18) (Table 4).

Ethical leadership’s mediation effect, however, is weaker for perceived CSR practices, which have a strong total effect on the three SWB indicators (Table 5). This impact was reduced after ethical leadership was introduced into the analyses. Overall, a significant mediation effect was observed only for positive feelings (B = 0.59; 95% CI = 0.10, 1.09), while the indirect effects on negative feelings (B = −0.28; 95% CI = −0.75, 0.22) and life satisfaction (B = 0.13; 95% CI = −0.06, 0.32) are statistically nonsignificant.

**Figure 2:**
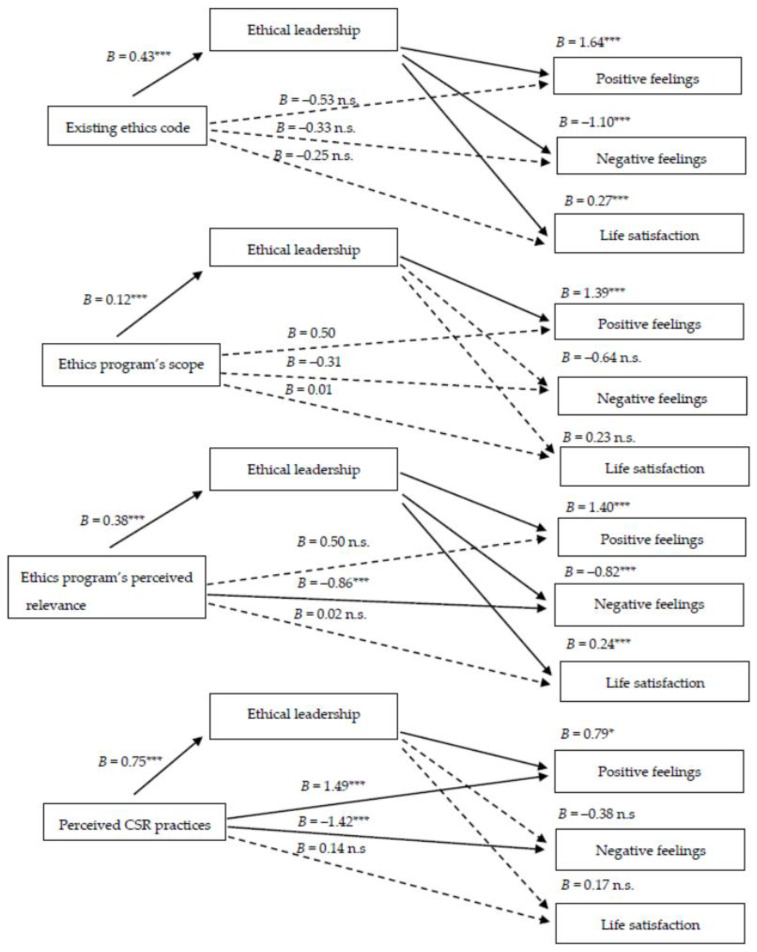
Mediation analysis: summary of results. Note: B, non-standardized coefficients (direct effects). * *p* < 0.05; *** *p* < 0.001; n.s., non-significant.

In short, the findings for ethical leadership’s mediation effect on the relationship between internal ethical contexts and workers’ SWB partially support hypothesis H2. Leaders’ ethical behavior mediates 9 of the 12 pairs of relationships analyzed. Figure 2 summarizes the results.

## 5. Discussion

### 5.1. Main Findings

The present study examined how the internal ethical context’s components are related to workers’ SWB and if ethical leadership serves as a mediating variable in the relationship between the internal ethical context’s components and SWB. The above results indicate that overall, organizations’ internal ethical context tends to have a positive effect on their employees’ SWB despite some heterogeneous findings for specific context elements. For instance, the mere existence of an ethics code or similar guidelines does not improve SWB among workers. This result is in line with previous research that has found that this kind of formal document’s existence is not enough to generate differences in individuals’ behavior [33,86]. However, along with other organizational variables, ethics codes can have an important impact on employees’ emotional, attitudinal, and behavioral responses. The present study’s results specifically provide clear evidence that an existing ethics code in organizations contributes to workers’ positive feelings and life satisfaction only when codes are combined with strong ethical leadership.

The findings also indicate that the broader the ethics program’s scope is, the less likely employees are to experience negative emotions in the workplace, but no direct effect was detected for the remaining SWB indicators. In contrast, if ethical leadership is considered, the broader the ethics programs’ scope is, the stronger the latter variable’s favorable impact on positive feelings and life satisfaction becomes. Similarly, the importance that staff members give to their ethics program affects their SWB, directly influencing the frequency of their positive and negative feelings. These effects are again mediated by ethical leadership—a path by which ethics programs’ relevance indirectly influences life satisfaction. The results thus reveal that an existing ethics code and ethics programs’ scope and relevance mostly contribute indirectly to employees’ well-being via ethical leaders’ intervention.

The findings for perceived CSR practices are quite distinct. This variable significantly affects all indicators of workers’ well-being and ethical leadership. That is, organizations’ socially responsible practices communicate how much they value and respect their employees, who then have greater SWB and leaders with more appropriate ethical conduct. However, ethical leadership can only partially explain CSR’s ability to increase positive feelings since this leadership style’s mediation effect is nonsignificant for negative feelings and life satisfaction.

CSR practices’ direct effect on employees’ SWB is in line with recent research [71,87] that implies that CSR is an ally of more traditional ethics programs in terms of promoting SWB. Prior studies have found support for a positive link between employee-focused CSR and ethical human resource management practices [88]. Although the current research proposed and tested ethical leadership’s role as a mediator, this variable might have a different relationship with perceived CSR, as indicated by some studies that have explored the two variables’ interactive effects on employees’ behavior [89,90].

The present results extend the existing evidence that ethical leadership is influenced by an organization’s internal ethical context [31,63] and contributes to influencing workers’ well-being [14,15,65,67,75]. The current results also suggest that ethical leadership plays a crucial role in highlighting and embodying organizations’ ethical standards and orientation, thereby influencing employees’ SWB. The findings thus indicate that internal ethical contexts are an important source of support for SWB. More specifically, organizations that favor ethical behavior and incorporate internal ethical context elements into their activities can enhance their staff members’ SWB. Organizations need to pay more attention to ethical issues, implementing a broad ethics program that workers can see is an important part of daily activities. Employees will then feel happier and more satisfied with their lives, especially if leaders act ethically and encourage conduct in line with their organization’s ethical guidelines.

### 5.2. Theoretical and Practical Implications

These results strengthen the literature on business ethics and well-being at work by extending the existing knowledge in an important new direction. The findings suggest that internal ethical context not only prevents harm within organizations but also fosters positive outcomes in employees’ SWB, thereby expanding the literature on ethical contexts’ influence. The present results in combination with recent research imply that organizational ethics promote employees’ well-being—as measured by indicators of reduced work-related stress [15,75]—and have a positive effect on life satisfaction via employees’ work life quality [14]. Internal ethical context factors produce dissimilar results regarding SWB indicators since these factors appear to influence SWB’s affective dimension (positive and negative feelings) more directly than its cognitive dimension (life satisfaction). The current results thus provide additional support for SWB’s bi-dimensionality [17].

One of the most important findings of the present study contributes to the literature on ethical leadership by highlighting the crucial role of leaders’ ethical behavior as a key leverage point for internal ethical context factors’ impact on followers’ affective and cognitive well-being. These results have managerial implications, suggesting that decision makers, managers, and human resource professionals should value, implement, and nurture a set of practices and infrastructure components that safeguard and promote organizations’ ethics. For instance, managers can create an environment conducive to ethical attitudes and behavior by supporting a broad ethics program that must be communicated and shared with all employees, including sanction and reward mechanisms.

Acting as role models, in accordance with ethical values and principles [56], leaders appear to be an extremely important source of support for successful coping appraisal processes, which in turn are essential to employees’ internalization of ethical values and ethics programs’ overall effectiveness as well as reducing ethical ambiguity and work-related stress [75]. Therefore, organizations need to acknowledge that leaders’ commitment to supporting ethical behaviors and CSR practices is a fundamental tenet of organizational ethics. In this context, CSR practices should include those related to workers’ needs and expectations, such as work–life balance, continuous learning, equity, and career development, which can also contribute to a fairer, healthier workplace and thus to individuals’ SWB.

### 5.3. Limitations and Suggestions for Future Research

Despite the additional knowledge the present results bring to the literature, this research’s limitations must be considered when interpreting and generalizing the findings. First, convenience sampling was used to facilitate the data collection. This method is often applied in research to gather data from naturally occurring groups within a given population (e.g., workers), but this sampling technique limits the results’ usefulness in other settings. Future research could use more representative samples of organizations’ staff members to ensure greater generalizability. Data heterogeneity issues should also be considered in future research, as they can pose a serious threat to the accuracy of the results.

Second, the correlational research design does not allow firm conclusions to be drawn about the causal nexus between the variables in question. This nexus can be established based on the relevant literature, but the models subjected to empirical analyses are statistically recursive. Further studies of this topic could adopt a longitudinal research design to reach more valid conclusions about causality.

Last, another limitation is due to the collection of cross-sectional data from a single source (CMV) [79,80]. The analyses’ results indicate that CMV is not a serious concern in the present research’s results, but future studies may want to adopt a time-lagged data collection strategy to reduce CMV’s potential occurrence more effectively.

Researchers may want to include additional variables in their conceptual model to understand more fully how organizations’ internal ethical context enhances employees’ SWB. Plausible mediating variables others than ethical leadership could also be incorporated. For instance, ethical context’s components may generate stronger perceptions of organizational justice or trust in organizations, which then stimulates more SWB among employees.

Future research can also explore if organizations’ ethical context differently influences the SWB levels of workers at different job positions (e.g., blue versus white collar) and types of organization (e.g., public versus private). As the ethical requirements and expectations might be dissimilar, the resultant levels of SWB might vary.

## 6. Conclusions

Overall, the current findings contribute significantly to the fields of business ethics and SWB by providing evidence that organizations’ internal ethical context—with leaders’ support—not only prevents misconduct but also fosters their staff members’ welfare.

Additional evidence of the internal ethical context’s influence on SWB could lead to purely utilitarian inferences given the already widely acknowledged association between SWB and various desirable organizational outcomes [18]. The latter include job attitudes and individual performance, which contribute to organizational success [16,22,91]. However, SWB must also be seen by both organizations and individuals as a superordinate value that everyone should esteem and nurture in order to ensure alignment with basic human rights and sustainable development goals. By creating ethical, socially responsible workplaces, organizations are ultimately fulfilling their duty as corporate citizens and contributing to healthier, happier societies.

## Figures and Tables

**Table 1:** Means, standard deviations, Pearson’s correlations, Cronbach’s alphas, squared correlations, composite reliability, and average variance extracted estimates.

Variables	M	SD	1	2	3	4	5	6	7	8	CR	AVE
1. Existing ethics code (0 = no; 1 = yes)	–	–	–								–	–
2. Ethics program’s scope	3.63	1.68	–	–							–	–
3. Ethics program’s perceived relevance	3.36	0.84	0.39 **	0.50 **	(0.72)	0.14	0.14	0.06	0.07	0.01	0.76	0.52
4. Perceived CSR practices	3.72	0.72	0.20 **	0.30 **	0.37 **	(0.86)	0.40	0.18	0.12	0.03	0.86	0.48
5. Ethical leadership	3.72	0.86	0.22 **	0.25 **	0.37 **	0.63 **	(0.95)	0.14	0.07	0.03	0.95	0.66
6. Positive feelings	22.14	3.62	0.02	0.14	0.24 **	0.42 **	0.37 **	(0.90)	0.36	0.27	0.90	0.60
7. Negative feelings	14.61	3.62	−0.10	−0.18 *	−0.27 **	−0.34 **	−0.27 **	−0.60 **	(0.82)	0.12	0.80	0.49
8. Life satisfaction	4.92	1.20	−0.05	0.05	0.08	0.16 *	0.18 **	0.52 **	−0.34 **	(0.84)	0.85	0.53

Notes: M, mean; SD, standard deviation; CR, composite reliability; AVE, average variance extracted; * *p* < 0.05; ** *p* < 0.01; Cronbach’s alphas between parentheses and squared correlations above the diagonal.

**Table 2:** Effects of existing formal ethics code or similar guidelines on subjective well-being (total, direct, and indirect effects).

Variables	Ethical Leadership B (SE)	Positive Feelings B (SE)	Negative Feelings B (SE)	Life Satisfaction B (SE)	Conclusion
Total effects					
Constant	–	22.00 (0.50) ***	15.23 (0.50) ***	5.02 (0.16) ***	
Ethics code	–	0.18 (0.57)	−0.81 (0.57)	−0.13 (0.19)	H1a not supported
Direct effect					
Constant	0.38(0.12) ***	16.46 (1.02) ***	18.94 (1.07) ***	4.09 (.36) ***	
Ethics code	0.43 (0.13) ***	−0.53 (0.54)	−0.33 (0.56)	−0.25 (0.19)	
Ethical leadership	–	1.64 (0.27) ***	−1.10 (0.28) ***	0.27 (0.09) ***	
Indirect effect					
B (SE)	–	0.71 (0.28)	−0.48 (0.22)	0.12 (0.06)	
(95% CI)	–	(0.22, 1.30)	(−0.98, −0.12)	(0.02, 0.26)	H2a supported
*R* ^2^	0.05	0.17	0.07	0.04	
F (df)	F (1, 220) = 10.81; *p* < 0.001	F (2, 219) = 18.42; *p* < 0.001	F (2, 219) = 8.70; *p* < 0.001	F (2, 219) = 4.42; *p* < 0.01	

Notes: *** *p* < 0.001; B, non-standardized coefficients; SE, standard error; CI, confidence interval; F (df), F-distribution (degrees of freedom).

**Table 3:** Effects of the ethics program’s scope on subjective well-being (total, direct, indirect effects).

Variables	Ethical Leadership B (SE)	Positive Feelings B (SE)	Negative Feelings B (SE)	Life Satisfaction B (SE)	Conclusion
Total effects					
Constant	–	21.04 (0.69) ***	15.81 (0.64) ***	4.76 (0.23) ***	
EPS	–	0.32 (0.18) ^+^	−0.38 (0.16) *	0.04 (0.06)	H1b partially supported
Direct effect					
Constant	3.38(0.14) ***	16.35 (1.37) ***	17.96 (1.33) ***	3.97 (0.74) ***	
EPS	0.12 (0.04) ***	0.15 (0.17)	−0.31 (0.17) ^+^	0.01 (0.06)	
Ethical leadership	–	1.39 (0.35) ***	−0.64 (0.35) ^+^	0.23 (0.12) ^+^	
Indirect effect					
B (SE)	–	0.17 (0.07)	−0.08 (0.05)	0.03 (0.02)	
(95% CI)	–	(0.05, 0.34)	(−0.20, 0.01)	(0.00, 0.07)	H2b partially supported
*R* ^2^	0.06	0.10	0.05	0.02	
F (df)	F (1, 166) = 11.35; *p* < 0.001	F (2, 165) = 9.58; *p* < 0.001	F (2, 165) = 4.60; *p* < 0.01	F (2, 165) = 2.07; n.s.	

Notes: ^+^ *p* < 0.10; * *p* < 0.05; *** *p* < 0.001; B, non-standardized coefficients; SE, standard error; EPS, ethical program’s scope; CI, confidence interval; F (df), F-distribution (degrees of freedom); n.s., not significant.

**Table 4:** Effects of the ethics program’s perceived relevance on subjective well-being (total, direct, and indirect effects).

Variables	Ethical Leadership B (SE)	Positive Feelings B (SE)	Negative Feelings B (SE)	Life Satisfaction B (SE)	Conclusion
Total effects					
Constant	–	18.69 (0.97) ***	18.53 (.97) ***	4.54 (0.33) ***	
EPR	–	1.03 (0.28) ***	−1.17 (0.28) ***	0.11 (0.10)	H1c partially supported
Direct effect					
Constant	2.45 (0.22) ***	15.26 (1.16) ***	20.55 (1.19) ***	3.95 (0.41) ***	
EPR	0.38 (0.06) ***	0.50 (0.29) ^+^	−0.86 (0.30) ***	0.02 (0.10)	
Ethical leadership	–	1.40 (0.28) ***	−0.82 (0.29) ***	0.24 (0.10) *	
Indirect effect					
B (SE)	–	0.52 (0.15)	−0.31 (0.14)	0.09 (0.04)	
(95% CI)	–	(0.26, 0.86)	(−0.62, −0.08)	(0.01, 0.18)	H2c supported
*R* ^2^	0.14	0.15	0.11	0.03	
F (df)	F (1, 220) = 34.58; *p* < 0.001	F (2, 219) = 19.62; *p* < 0.001	F (2, 219) = 13.05; *p* < 0.001	F (2, 219) = 3.56; *p* < 0.05	

Notes: ^+^ *p* < 0.10; * *p* < 0.05; *** *p* < 0.001; B, non-standardized coefficients; SE, standard error; EPR, ethics program’s relevance; CI, confidence interval; F (df), F-distribution (degrees of freedom).

**Table 5:** Effects of perceived corporate social responsibility practices on subjective well-being (total, direct, and indirect effects).

Variables	Ethical Leadership B (SE)	Positive Feelings B (SE)	Negative Feelings B (SE)	Life Satisfaction B (SE)	Conclusion
Total effects					
Constant	–	14.40 (1.16) ***	20.97 (1.20) ***	3.91 (0.42) ***	
CSR	–	2.08 (0.31) ***	−1.17 (0.32) ***	0.27 (0.11) **	H1d supported
Direct effect					
Constant	0.94 (0.24) ***	13.65 (1.19) ***	21.33 (1.25) ***	3.75 (0.43) ***	
CSR	0.75 (0.06) ***	1.49 (0.39) ***	−1.42 (0.11) ***	0.14 (0.14)	
Ethical leadership	–	0.79 (0.33) *	−0.38 (0.34)	0.17 (0.12)	
Indirect effect					
B (SE)	–	0.59 (0.20)	−0.28 (0.25)	0.13 (0.10)	
(95% CI)	–	(0.10, 1.09)	(−0.75, 0.22)	(−0.06, 0.32)	H2d partially supported
*R* ^2^	0.39	0.19	0.12	0.04	
F (df)	F (1220) = 143.14; *p* < 0.001	F (2219) = 26.30; *p* < 0.001	F (2219) = 15.07; *p* < 0.001	F (2219) = 4.07; *p* < 0.05	

Notes: ** *p* < 0.01; * *p* < 0.05; *** *p* < 0.001; B, non-standardized coefficients; SE, standard error; CI, confidence interval; F (df), F-distribution (degrees of freedom).

## Data Availability

The data will be made available on a reasonable request by contacting the corresponding author.
